# Urinary bladder pyogenic granuloma: a case report

**DOI:** 10.1186/1752-1947-6-149

**Published:** 2012-06-12

**Authors:** Shoichiro Mukai, Hiroyuki Tanaka, Kouji Yamasaki, Takayuki Goto, Chie Onizuka, Toshiyuki Kamoto, Hiroaki Kataoka

**Affiliations:** 1 Department of Urology, Faculty of Medicine, University of Miyazaki, 5200 Kihara, Kiyotake, Miyazaki, Japan; 2 Section of Oncopathology and Regenerative Biology, Faculty of Medicine, University of Miyazaki, 5200 Kihara, Kiyotake, Miyazaki, Japan

## Abstract

**Introduction:**

Although more than 100 cases of hemangioma of the urinary bladder have been reported, capillary-type hemangiomas of the bladder are rare. Pyogenic granulomas, which are common tumor-like vascular lesions of the skin and oral mucous membranes, reveal histopathological findings similar to capillary-type hemangiomas and are differentiated from ordinary hemangiomas by clinical features and etiologic factors. Little is known regarding the occurrence of pyogenic granulomas in the urinary bladder.

**Case presentation:**

We present the case of a 78-year-old Japanese man who had developed a hemangiomatous lesion in his bladder which led to acute clot retention. He had a recent history of chemotherapy for pancreatic cancer. A solitary pedunculated mass measuring 1.2 cm was observed in the bladder. Histopathological analysis of the resected mass revealed marked lobular capillary proliferation with surface erosions.

**Conclusion:**

Cystoscopic and pathologic findings in addition to possible predisposing factors supported a diagnosis of pyogenic granuloma of the urinary bladder.

## Introduction

Although vascular tumors, especially hemangiomas, are relatively common mesenchymal tumors in soft tissue, skin and organs such as the liver, hemangioma of the urinary bladder is a rare tumor that accounts for only 0.6% of all bladder tumors [1]. Hemangiomas have been classified into two histologic types, cavernous, the most common, and capillary. In addition, arteriovenous hemangiomas (arteriovenous malformation) have been reported as a histologic variant [1-3]. These tumors are usually small, and when they occur in the bladder, the most common presenting symptom is mild hematuria [1-3]. Cystoscopic findings vary from small punctuate areas and slightly raised lesions to polypoid formations located on the posterior or lateral wall, or dome of the bladder [1-3]. Transurethral endoscopic surgery is the most common treatment and usually yields a favorable outcome [1-3].

Pyogenic granuloma is a common tumor-like vascular lesion of the skin and mucous membranes of the oral cavity that has traditionally been considered a reactive hyperplastic lesion rather than a neoplastic process [4]. It usually presents as a solitary, rapidly growing papule or polyp-like pedunculated lesion that bleeds easily because of its extreme vascularity and eroded surface [4-6]. Pyogenic granuloma has been histologically classified as a lobular capillary hemangioma, which is similar to a capillary-type hemangioma. We report the case of a patient with a hemorrhagic, pedunculated polyp-like bladder lesion with macroscopic and histologic features consistent with a diagnosis of pyogenic granuloma of the urinary bladder.

## Case presentation

A 78-year-old Japanese man was referred for sudden, asymptomatic gross hematuria with pyuria and clot retention that had occurred six days prior to his visit. Upon presentation at our hospital, he was afebrile and denied chills, pain, or weight change. Physical examination revealed no palpable mass or enlarged lymph nodes. The patient had undergone transurethral resection of the prostate for benign prostatic hyperplasia and transurethral lithotripsy for bladder calculi 11 years previously. He had also been diagnosed with pancreatic cancer (non-functional malignant endocrine tumor, pT2N3M1) seven years previously. Following resection of the pancreatic tumor, he had received chemotherapy for liver and lymph node metastases. Five cycles of gemcitabine therapy proved ineffective; however, stable disease was achieved by second-line chemotherapy (administered until 17 months prior to his being seen for urinary symptoms) using tegafur, gimeracil, oteracil potassium and octreotide. Because of severe adverse events (nausea, appetite loss), monotherapy using octreotide and best supportive care had been provided.

**Figure 1:**
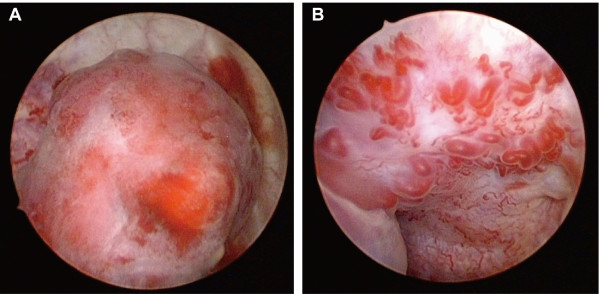
(**A**)**:** Cystoscopy revealed a red pedunculated mass with small surface ulcerations. (**B**): The stalk of the tumor contains vessels originating from the surrounding mucosa.

**Figure 2:**
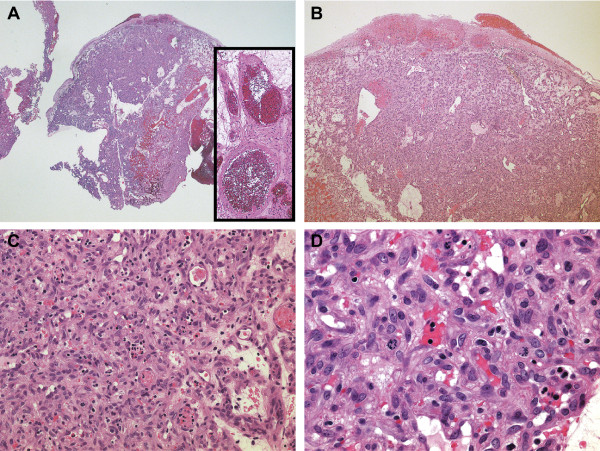
(**A**): Low-magnification image (×1.25) shows proliferation of capillary-sized vessels in a lobular arrangement. Larger dilated vessels are observed at the base of the stalk (inset). (**B**): Surface is eroded (×4). (**C**): Anastomosing small blood vessels are lined with plump endothelial cells. The stroma is edematous with mild infiltration of mononuclear cells and neutrophils (×200). (**D**) Mitoses are occasionally seen in the endothelial cells (×400).

Cystoscopy revealed a solitary non-papillary, pedunculated mass measuring approximately 1.2 cm located lateral to the left ureteral orifice (Figure 1A). The surface of the mass appeared reddish and highly vascular. Distended vessels were observed at the base of the mass originating from the surrounding mucosa, indicating that they were feeding vessels (Figure 1B). Transurethral resection of the mass was performed and the tissue was submitted for histopathological examination. Magnetic resonance imaging and cystoscopy four months after surgery did not reveal any residual tumor. The pedunculated lesion had an eroded surface and was composed of a lobular proliferation of capillary-sized vessels with large dilated vessels, possibly feeding vessels, at the base of the lesion (Figure 2A, B). Variable anastomosing small blood vessels admixed with dilated and sinusoidal vessels were lined with plump endothelial cells (Figure 2C). The endothelial cells were positive for CD31, CD34, and factor VIII-related antigen, but negative for podoplanin (antibody D2-40) (data not shown). While mitoses were occasionally observed, the endothelial cells were without atypia (Figure 2D). Edematous and/or fibromyxoid changes were observed in the stroma. Mild lymphoplasmacytic infiltration with intermingled neutrophils was seen. Therefore, the histopathologic findings were consistent with a capillary hemangioma with eroded surface. Because of the acute symptoms together with the cystoscopic and histologic findings, we believe that the lesion should be considered a pyogenic granuloma of the bladder.

## Discussion

Hemangiomas of the urinary bladder are the second most common vascular tumor of the urinary tract, with more than 100 cases reported [1-3,7,8]. Hemangiomas have traditionally been classified as cavernous or capillary, with some authors arguing for inclusion of an arteriovenous type [3]. Most hemangiomas are cavernous types. Chen *et al*. reported that bladder hemangiomas occur in all age groups, with most being diagnosed in patients less than 30 years of age at a male-to-female ratio of 3.7:1 [1]. The tumors usually present as an incidental finding during evaluation for hematuria with or without voiding symptoms [1-3,7,8]. In addition, hemangiomas of childhood are seen in congenital syndromes, namely, the Klippel-Trénaunay-Weber syndromes, which have multiple hemangiomas [2,3,7,8].

On cystoscopy, bladder hemangiomas most commonly appear as elevated sessile blue masses. In our patient, however, we observed a red pedunculated tumor with probable large feeding vessels in the stalk. Similar gross findings have been observed in pyogenic granulomas of the oral cavity, skin, and gastrointestinal lesions [4-6]. Although cystoscopic findings of hemangioma can be varied, we were not able to find macroscopic findings similar to ours in other reported bladder hemangiomas [1-3,7,8].

A marked pathological finding of pyogenic granulomas is prominent capillary growth within a hyperplastic granulation reaction, which suggests strong angiogenic activity. Similar histopathology can be seen in capillary hemangiomas and pyogenic granulomas, and there are no differential findings or available diagnostic markers to determine the angiogenic profile and an exact differential diagnosis [9]. Clinical features and etiologic factors may be different in hemangioma and pyogenic granuloma because the former is a neoplastic lesion and the latter is considered to be a reactive lesion. In the current case, hematuria of sudden onset was the initial symptom. Pyuria was also seen and the patient had also previously undergone chemotherapy, a possible etiologic factor for this lesion. The clinical observations favored a diagnosis of pyogenic granuloma over capillary hemangioma.

Pyogenic granuloma of the urinary bladder is very rare; there has only been one report of seven cases, which was published as an abstract in 1973 without additional information [10]. In the current case, the pathological findings were consistent with both capillary hemangioma and pyogenic granuloma. However, the macroscopic (cystoscopic) findings and clinical history supported a diagnosis of pyogenic granuloma of the urinary bladder, the first case to be reported since the above-mentioned abstract. Further investigation to differentiate between capillary hemangioma and pyogenic granuloma of the urinary bladder may be warranted.

There are many therapeutic approaches for the management of patients with hemangioma. Partial cystectomy, simple cystectomy with bladder augmentation, and radiation therapy are effective for bulky and multiple tumors. However, endoscopic biopsy and fulguration, and yttrium aluminium garnet laser coagulation have become standard in cases of small tumors [1-3]. In the present case, transurethral resection was performed and the patient remained well for more than four months following surgery, with no evidence of recurrence.

## Conclusions

Pyogenic granuloma commonly occurs on the skin and oral mucosa, but is extremely rare in the urinary bladder. We treated a 78-year-old Japanese man with a capillary hemangioma-like lesion of the urinary bladder that resulted in clot retention. Cystoscopic and pathologic findings together with his clinical history led to the conclusion that the lesion was a urinary bladder counterpart of pyogenic granuloma usually found in skin and oral mucosa.

## Consent

Written, informed consent was obtained from the patient for publication of this case report and accompanying images. A copy of the written consent is available for review by the Editor-in-Chief of this journal.

## Competing interests

The authors declare that they have no competing interests.

## Author’s contributions

SM drafted the report, performed the surgical procedure and approved the final version of the manuscript. CO, KY, TG and TK performed examinations, cared for the patient, and approved the final version of the manuscript. HK reviewed the pathological specimens with HT, drafted the report and contributed to the final version of the manuscript. All authors read and approved the final manuscript

## References

[B1] Chen L Nascimento AG Neumann RM Nehra A Cheville JC Ramnani DM Leibovich BC Bostwick DG Hemangioma of the urinary bladder Cancer 1999 86 498 504 10.1002/(SICI)1097-0142(19990801)86:3<498::AID-CNCR19>3.0.CO;2-6 10430259

[B2] Lott S Lopez-Beltran A Maclennan GT Montrioni R Cheng L Soft tissue tumors of the urinary bladder, part I: myofibroblastic proliferations, benign neoplasms, and tumors of uncertain malignant potential Hum Pathol 2007 38 807 823 10.1016/j.humpath.2007.03.017 17509394

[B3] Tavora F Montgomery E Epstein JI A series of vascular tumors and tumorlike lesions of the bladder Am J Surg Pathol 2008 32 1213 1219 10.1097/PAS.0b013e31816293c5 18580491

[B4] Neville BW Damm DD Allen CM Bouquot JE Oral and Maxillofacial Pathology 2008 3 W.B Saunders, Philadelphia 517 519

[B5] Zaballos P Carulla M Ozdemir F Zalaudek I Bañuls J Llambrich A Puig S Argenziano G Malvehy J Dermoscopy of pyogenic granuloma: a morphological study Br J Dermatol 2010 163 1229 1237 10.1111/j.1365-2133.2010.10040.x 20846306

[B6] Moffatt DC Warwryko P Singh H Pyogenic granuloma: An unusual cause of massive gastrointestinal bleeding from small bowel Can J Gastoenterol 2009 23 261 264 10.1155/2009/579163 PMC2711675 19373418

[B7] Wiygul JB Palmer L Isolated hemangioma causing gross painless hematuria in an adolescent male Urology 2010 76 463 464 10.1016/j.urology.2009.11.055 20163837

[B8] Ashley RA Figueroa TE Gross hematuria in a 3-year-old girl caused by a large isolated bladder hemangioma Urology 2010 76 952 954 10.1016/j.urology.2010.03.062 20627282

[B9] Freitas TM Miguel MC Silveria EJ Freitas RA Galvão HC Assessment of angiogenic markers in oral hemangiomas and pyogenic granulomas Exp Mol Pathol 2005 79 79 85 10.1016/j.yexmp.2005.02.006 16005715

[B10] Anderson CK Pyogenic granuloma of the urinary bladder J Clin Pathol 1973 26 984 4784512 10.1136/jcp.26.12.984-b PMC477961

